# Evaluation of candidate reference genes for RT-qPCR studies in three metabolism related tissues of mice after caloric restriction

**DOI:** 10.1038/srep38513

**Published:** 2016-12-06

**Authors:** Huan Gong, Liang Sun, Beidong Chen, Yiwen Han, Jing Pang, Wei Wu, Ruomei Qi, Tie-mei Zhang

**Affiliations:** 1 The MOH key laboratory of Geriatrics, Beijing Hospital, National Center of Gerontology, Beijing, P.R. China

## Abstract

Reverse transcription quantitative-polymerase chain reaction (RT-qPCR) is a routine method for gene expression analysis, and reliable results depend on proper normalization by stable reference genes. Caloric restriction (CR) is a robust lifestyle intervention to slow aging and delay onset of age-associated diseases via inducing global changes in gene expression. Reliable normalization of RT-qPCR data becomes crucial in CR studies. In this study, the expression stability of 12 candidate reference genes were evaluated in inguinal white adipose tissue (iWAT), skeletal muscle (Sk.M) and liver of CR mice by using three algorithms, geNorm, NormFinder, and Bestkeeper. Our results showed β2m, Ppia and Hmbs as the most stable genes in iWAT, Sk.M and liver, respectively. Moreover, two reference genes were sufficient to normalize RT-qPCR data in each tissue and the suitable pair of reference genes was β2m-Hprt in iWAT, Ppia-Gusb in Sk.M and Hmbs-β2m in liver. By contrast, the least stable gene in iWAT or Sk.M was Gapdh, and in liver was Pgk1. Furthermore, the expression of Leptin and Ppar-γ were profiled in these tissues to validate the selected reference genes. Our data provided a basis for gene expression analysis in future CR studies.

Quantification of gene expression has become a staple in most molecular biological laboratories to understand the complex signaling networks that regulate different responses observed under various conditions. Reverse transcription quantitative real-time polymerase chain reaction (RT-qPCR) has become the most prevalent and powerful technique applied to quantify gene expression because of its simple, sensitive, accurate, reproducible and high-throughput features^1^. RT-qPCR gives a rapid means of understanding gene transcriptional level variation under various conditions. There are two methods of presenting quantitative gene expression - the absolute and relative quantification methods^1^,^2^. For researchers, it is often necessary to know not the actual copy number of gene but the expression change of gene, so that, relative quantification method is more common for gene expression assessment^1^,^3^. However, relative quantification presents qPCR data of target genes relying on internal control genes as reference^4^.

Reference genes with stable expression levels are essential as an internal control to normalize and monitor the variations of interested gene expression among different treatments or conditions^5^,^6^. The ideal reference genes should be expressed at constant levels regardless of experimental conditions, cell types, tissues, developmental stages or stress treatments^4^,^7^, and should have expression levels comparable to that of the target gene^8^. Nevertheless, increasing evidences suggest that the expression of reference genes often varies considerably under different experimental conditions as reviewed by Suzuki and Kozera^9^,^10^. Thus, identification of reliable reference genes is a prerequisite for qRT-PCR experiments. However, it has been ignored in most of the RT-qPCR studies that it is necessary to validate the stability of the reference gene under determined experimental condition to ensure proper normalization and a robust RT-qPCR analysis^6^,^10^,^11^.

Caloric restrictions (CR) is an efficient non-genetic intervention to slow aging, improve health, reduce most risk factors for chronic diseases and delay onset of age-associated diseases in a fashion that is evolutionarily conserved from yeast to primates and humans^12^,^13^,^14^,^15^,^16^. CR causes global changes in gene expressions which are the molecular basis of the beneficial effects of CR^15^,^17^,^18^. To decipher the mechanism of CR further and achieve the beneficial effects of CR easier, it’s necessary and important to analyze the changes of gene transcriptional level. The work in rat cortex and hippocampus showed that CR significantly influenced expression of 18S rRNA and cyclophilin B^19^. However, to date, appropriate reference genes have not been identified in case of CR in other tissues or organs, especially those tissues which are closely related to metabolism and have extensively response to CR, such as liver, skeletal muscle and white adipose tissue^16^,^20^,^21^. It has been reported that in adipose tissue or adipocyte, some of the commonly-used reference genes, for instance glyceraldehyde-3-phosphate dehydrogenase (GAPDH), can be regulated by some treatments, such as insulin or obesity^22^,^23^,^24^. Thus, it is essential to evaluate the stability of reference genes in these tissues after CR.

Several software packages have been established to identify reference genes with stable expression levels, such as geNorm^7^, NormFinder^25^, and BestKeeper^26^. With the help of these statistical algorithms, a number of studies on validation of reference genes have been reported^27^,^28^,^29^. In this study, we performed the first comprehensive study of CR effects in inguinal white adipose tissue (iWAT), skeletal muscle (Sk.M) and liver on twelve commonly-used reference genes involved in different biological functions^9^,^27^,^30^,^31^,^32^,^33^,^34^ (Table 1), including the formation of cellular cytoskeleton (beta-actin (Actb) and alpha-tubulin (Tuba)), protein biosynthesis (hydroxymethylbilane synthase (Hmbs), peptidylprolyl isomerase A (Ppia) and ribosomal protein L13A (Rpl13a)), immune response (beta-2 microglobulin (β2m)), metabolism (Gapdh, beta-glucuronidase (Gusb) and phosphoglycerate kinase 1(Pgk1)), nucleotide synthesis (hypoxanthine guanine phosphoribosyl transferase (Hprt)), transcription (TATA box binding protein (Tbp)) and signal transduction (tyrosine 3-monooxygenase/tryptophan 5-monooxygenase activation protein, zeta-polypeptide (Ywhaz)). This study provides a basis for the selection of reference genes and useful guidelines for future gene expression studies of CR and metabolism.

## Materials and Methods

### Ethics statement

This study was carried out in strict accordance with the recommendations in the Guide for the Care and Use of Laboratory Animals of the National Institutes of Health. The protocol was approved by the Biomedical Ethics Committee of Beijing Hospital and Beijing Institute of Geriatrics, Beijing, China. Staff veterinarians monitored mice on a regular basis, finding no pathogens. All surgeries were performed under 10% chloral hydrate anesthesia, and all efforts were made to minimize suffering.

### Animals

Male C57BL/6 mice were purchased from the Vital River (Charles River China) at two months of age. After a one-week acclimation, all mice were randomly assigned to two groups and treated for 3 months: ad libitum (AL) fed mice were given uninhibited access to normal chow diet; caloric restriction (CR) mice were fed as 65~70% of the AL feeding rate^15^,^35^. They were housed individually. All the animals were housed at 21 °C in a 12-h light/12-h dark cycle. We recorded the body weight and food intake once a week during the study (Supplementary Fig. S1).

### IPGTT and IPITT

For intra-peritoneal glucose tolerance test (IPGTT), following an overnight fast, the mice were injected intraperitoneally of glucose at a dose of 1 g/kg body weight (10% glucose in PBS, 100 ul/10 g body weight)^36^. For intra-peritoneal insulin tolerance test (IPITT), following a 4 h fasting, mice were injected intraperitoneally of insulin at a dose of 0.75 IU/kg body weight (0.075IU/ml in PBS, 100 ul/10 g body weight)^36^. Blood glucose levels were measured from tail blood using glucose strips at 0 (basal), 30, 60 and 120 min after injection by One Touch Ultra glucose strips (LifeScan, Inc, USA).

### Tissue preparation

Mice were sacrificed according to the approved protocol. The liver, inguinal white adipose tissue (iWAT) and skeletal muscle (Sk.M) from hind legs were isolated, snap frozen in liquid nitrogen, ground into powder with mortar and pestle in liquid nitrogen and then stored at −80 °C.

### Total RNA extraction

Total RNA was isolated from 10–80 mg tissues using TRIzol® reagent (Invitrogen Life Technologies, USA) following the manufacturer’s instructions. The residual DNA was removed by TURBO DNA free kit (Ambion Inc., UK). Yield and purity of RNA was determined by NanoDrop ND-1000 spectrophotometer (Nanodrop technologies, USA). RNA samples with an absorbance ratio OD 260/280 between 1.9–2.2 and OD 260/230 greater than 2.0 were used for further analysis. RNA integrity was assessed using agarose gel electrophoresis.

### Reverse transcription cDNA synthesis

First-strand cDNA was synthesized from 2 μg of total RNA with random hexamer oligonucleotide primers using a 20 μl reverse transcription system (New England Biolabs, USA). cDNA was stored at −20 °C for future use. For qPCR analysis, each cDNA sample was diluted 20 times with nuclease free water.

### Real-time PCR

Real-time PCRs were conducted in Bio-Rad iQ5 Real- Time System. For each reaction, the 20 μl mixture contained 1 μl of diluted cDNA, 5 pmol each of the forward and reverse primers, and 10 μl 2 × SYBR Premix Ex Taq II (Takara Bio Inc, Japan). The amplification program was as follows: 95 °C for 30 s, 40 cycles at 95 °C for 5 s and 60 °C for 30 s. After amplification, a thermal denaturing cycle was added to derive the dissociation curve of the PCR product to verify amplification specificity (Supplementary Fig. S1). Reactions for each sample were carried out in triplicate.

qPCR efficiencies in the exponential phase were calculated for each primer pair using standard curves (5 tenfold serial dilutions of pooled cDNA that included equal amounts from the samples set), the mean threshold cycle (Ct) values for each serial dilution were plotted against the logarithm of the cDNA dilution factor and calculated according to the equation E = 10(−1/slope) × 100^11^, where the slope is the gradient of the linear regression line. The linear dynamic range was determined by the standard curve and correlation coefficients (R^2^) for each gene as reported.

### Primers

Twelve candidate reference genes were selected based on their common usage as endogenous control genes in previous studies. The candidate genes were Actb, β2m, Gapdh, Gusb, Hmbs, Hprt, Pgk1, Ppia, Rpl13a, Tbp, Tuba, Ywhaz. The primers were designed from nucleotide sequences identified using NCBI BLAST (http://blast.ncbi.nlm.nih.gov/Blast.cgi). Primers were ordered from Life Technologies with their certificates of analysis. The primer characteristics of nominated reference genes are listed in Table 2. The primers for validation of selected reference genes are as follows: Leptin-F, GTGGCTTTGGTCCTATCTGTC and Leptin-R, CGTGTGTGAAATGTCATTGATCC; Ppar-γ (peroxisome proliferator-activated receptor gamma) -F, TGTCGGTTTCAGAAGTGCCTTG and Ppar-γ-R, TTCAGCTGGTCGATATCACTGGAG.

### Analysis of gene stability and minimum number of reference genes required

To assess the stability of candidate reference genes, three widely recognized reference gene normalization algorithms each with a unique advantage were used, geNorm^7^, NormFinder^25^, and BestKeeper^26^. Ct values were converted to non-normalized relative quantities according to the formula: 2^−ΔCt^ (ΔCt = the corresponding Ct value – minimum Ct)^7^. geNorm and NormFinder calculations are based on these converted quantities; raw Cq values were directly analyzed by BestKeeper. The brief usage and characteristics of each algorithm are as follows:

GeNorm^7^ ranked the reference genes based on their M value. The lower the M value, the higher is the expression stability and the M value less than 1.5 is recommended to identify stably expressed gene. The two most stable reference genes are determined by step-wise exclusion of the least stable gene. It also compares the pairwise variation (V) of these genes with others. The V value of Vn/Vn + 1 between two sequential normalization factors was used to determine the optimal number of reference genes required for better normalization. A threshold value below 0.15 suggests the requirement of no additional reference gene for normalization.

NormFinder^25^ estimates both intra- and inter-group variations of gene expression and then combines the two to produce a stability value for all the samples in any number of groups. Genes with the lowest rank are considered to be most stably expressed and are ideal to select as reference gene(s) for that particular experimental condition.

BestKeeper is used to determine standard deviation and power of each reference gene, and then to select the best reference genes based on these variables^26^. The BestKeeper index is calculated from the geometric mean of the candidates Ct values for each specific sample. In BestKeeper, SD_Ct value_ < 1 indicates genes which are stably expressed. The most stable reference genes are the ones with the lowest SD values and highest coefficients of correlation with the BestKeeper index.

### Validation of reference genes

To confirm the reliability of the reference genes, the relative expression profiles of Leptin and Ppar-γ genes were determined and normalized with the recommended gene pair, most and least stable gene, respectively. Relative fold changes in gene expression were calculated using the comparative 2^−ΔΔCt^ method and normalized to the corresponding reference gene levels^1^,^37^.

### Statistical analysis

Data are expressed as mean ± standard error (s.e.m). Means of different groups were compared and analyzed using the Student’s t-test. Differences were reported as statistically significant when p < 0.05. GraphPad Prism 5 (GraphPad Software, USA) was used for statistical procedures and graph plotting.

## Results

### Establishment of CR mice model

**Figure 1:**
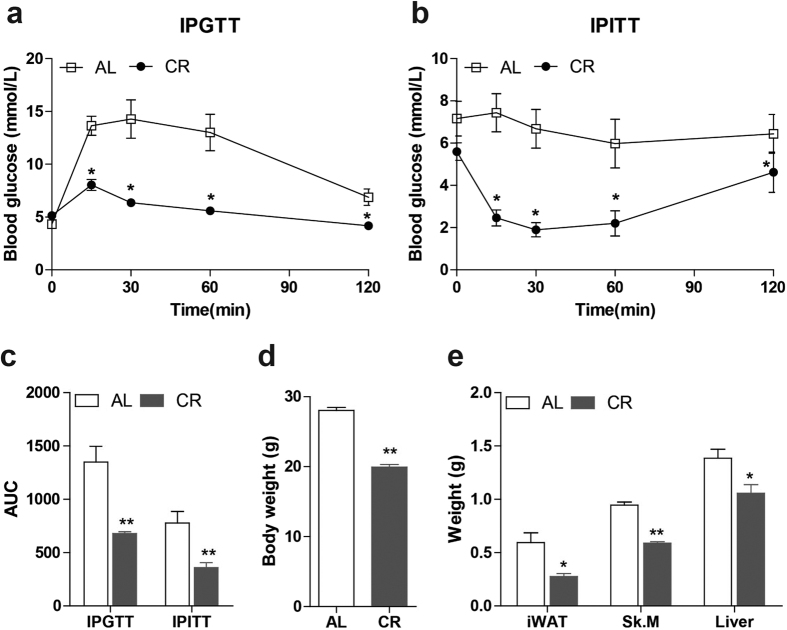
The assessment of CR model establishment. Glucose measurements (mmol/l) at 0, 30, 60 and 120 min after glucose (**a**) or insulin intraperitoneal injection (**b**) in mice after CR for 3 months. (**c**): AUC of IPGTT or IPITT was calculated accordingly. Weight of whole body (**d**), iWAT, Sk.M and liver (**e**) in both groups was measured after CR for 3 months. (*p < 0.05, **p < 0.01 vs AL. n = 5 in each group) iWAT: inguinal white adipose tissue, Sk. M: skeletal muscle, AUC: area under curve, IPGTT: intraperitoneal glucose tolerance test, IPITT: intraperitoneal insulin tolerance test, AL: ad libitum, CR: caloric restriction.

After treatment for 3 months, the body weight in CR group (19.94 ± 0.38 g) is significantly lower (p < 0.0001) than in AL group (28.03 ± 0.43 g) and is only 71.1% as in AL group (Fig. 1d). Since insulin sensitivity increase is one of the important effects of CR^14^,^38^,^39^, the establishment of CR was evaluated through assessing glucose and insulin homeostasis. We performed intraperitoneal glucose tolerance test (IPGTT) and insulin tolerance test (IPITT). Both glucose tolerance and insulin tolerance improved in CR mice relative to AL (Fig. 1a and b). Both of the area under the curve (AUC) measurements were significantly reduced in CR group (Fig. 1c. p = 0.002 and 0.008, respectively). Besides, the weight of iWAT, Sk.M and liver of CR mice reduced to 46.5% (p = 0.01), 62.2% (p < 0.0001) and 76.3% (p = 0.02) as AL mice, respectively. These results indicated that the CR model was established successfully.

### Expression profiles of candidate reference genes

Twelve candidate reference genes were selected for this study. They are widely used and recognized reference genes, which have been described in the literatures^9^,^27^,^30^,^31^,^32^,^33^,^34^ and represent several functional classes to minimize the possibility of co-regulation. The performance of each amplification primer set was tested by RT-qPCR. Their respective PCR amplification efficiencies were calculated as the first step. The amplification efficiency (E) of all reactions ranged from 96% to 107%; and the correlation coefficients (R^2^) of the standard curve varied from 0.9913 to 0.9999 (Table 2).

**Figure 2:**
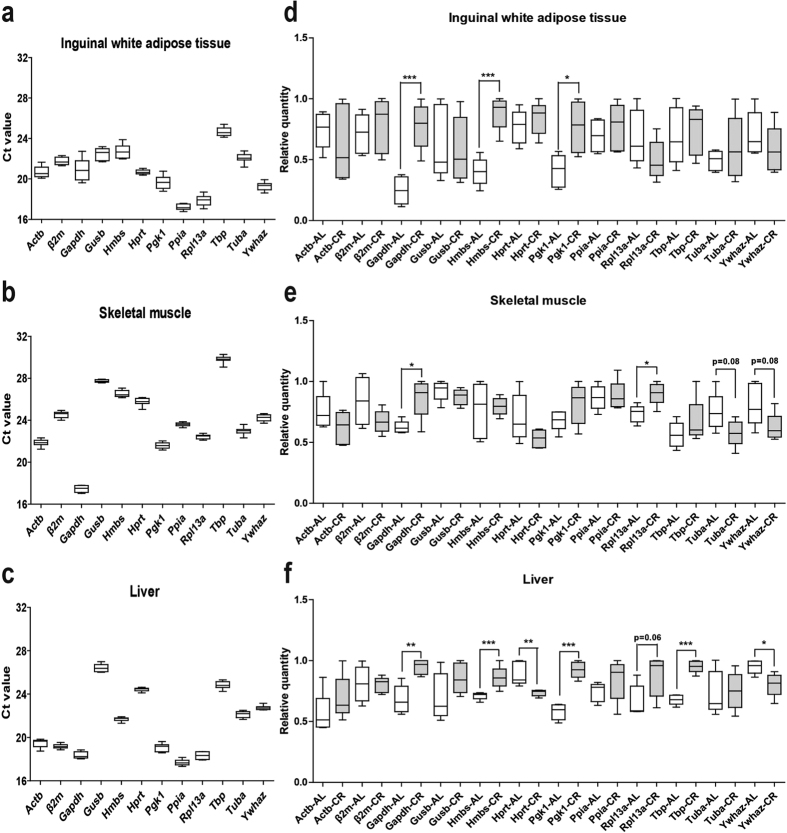
Ct values and relative quantities of 12 candidate reference genes in all samples of each tissue of CR mice. Raw Ct values and relative quantities (without normalization to any reference genes) of ten samples of inguinal white adipose tissue (**a** and **d**), skeletal muscle (**b** and **e**) or liver (**c** and **f**), including five AL and five CR mice were described using a box and whiskers plot. The boxes encompass the 25th to 75th percentiles, and the line across the box is the median. Whisker caps denote the maximum and minimum values. The non-normalized relative quantities were calculated by dCt method with iQ5 software (*p < 0.05, **p < 0.01 and ***p < 0.001 vs AL).

The expression profiles of candidate reference genes in all the three tissues of both groups were investigated (Fig. 2). The individual reference genes had different expression ranges across samples in each tissue. The expression levels of the candidate reference genes were determined by Ct values through RT-qPCR experiments. Ct is the amplification cycle number at which the fluorescence signal of the reporter dye reaches above an arbitrarily placed baseline threshold^1^. The distribution of the Ct values for each of the reference genes in different tissues is displayed by box plot. All the Ct values are in suitable range: 17.23 ± 0.10 to 24.67 ± 0.14 (standard deviation, SD is 0.30~1.07) in iWAT (Fig. 2a), 17.45 ± 0.11 to 29.81 ± 0.10 (SD is 0.18~0.35) in Sk.M (Fig. 2b) and 17.69 ± 0.08 to 26.42 ± 0.11 (SD is 0.18~0.38) in liver (Fig. 2c).

We measured non-normalized relative quantity (the raw expression without normalization to any reference gene) of our 12 genes (Fig. 2d–f) in iWAT, Sk.M and liver. Among the tested genes, we observed variations between AL and CR mice within all three tissues: there are significant differences in three genes (Gapdh, Hmbs and Hprt (p < 0.05)) in iWAT (Fig. 2d) and two genes (Gapdh and Rpl13a (p < 0.05)) in Sk.M (Fig. 2e), and more candidate reference genes were changed by CR in liver, including Gapdh, Hmbs Hprt, Rpl13, Tbp and Ywhaz (p < 0.05) (Fig. 2f). These results clearly demonstrate variations in many commonly-used reference genes, but also differences between different tissues changed by CR. It also indicates that one reference gene is usually not enough, and normalization to multiple reference genes should be done for each tissue during CR. To address this question, we used three different methods: geNorm, Normfinder and BestKeeper algorithms.

### Effect of CR on mRNA expression of candidate reference genes

**Figure 3:**
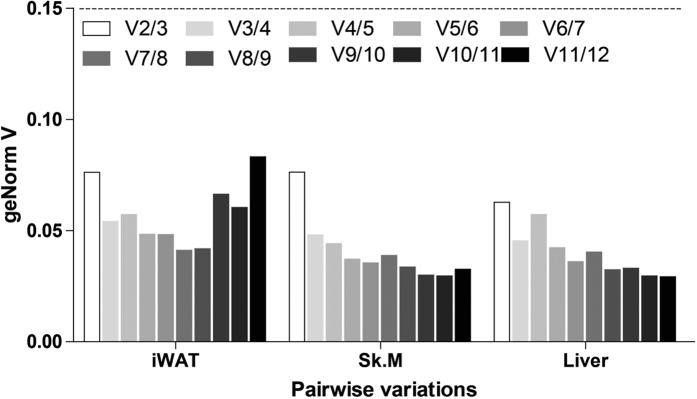
Pairwise variation (V) of candidate reference genes calculated by geNorm. Pairwise variation (Vn/n + 1) was analyzed to determine the optimal number of reference genes. Two reference genes are enough in all the detected tissues to normalize RT-qPCR data as the pairwise variation V value for V 2/3 (n/n + 1) is under 0.15. iWAT: inguinal white adipose tissue, Sk. M: skeletal muscle.

The candidate reference genes were ranked based on their gene expression stability between CR and AL treatments assessed by employing three statistical algorithms. All the geNorm V values generated by multiple analyses of pairwise variations between the twelve reference genes were under the 0.15 threshold in all these tissues (Fig. 3). It means that at least two reference genes among them are enough for normalization in RT-qPCR in each tissue. According to the geNorm algorithm, all candidate reference genes, regardless of which tissue, meet the programs default limit of M < 1.5 (0.251~1.024) (Table 3). Moreover, when considering the results of the BestKeeper algorithm, in which SD (±Ct) value < 1 (0.099~0.869) (Table 3) indicates that genes are stably expressed, these selected gene also were considered stable. However, the ranks order of the reference genes in each tissue showed similar trends with subtle variations by different software, which might be attributed to the differences across algorithms. For the purpose of clarity in depiction, we dealt separately the ranking of the reference genes in each tissue generated by different statistical method.

#### In inguinal white adipose tissue

In iWAT, it is showed that the best genes to use as reference genes were β2m > Tbp > Ppia recommended by geNorm, β2m > Ppia > Hprt by NormFinder and Hprt > β2m > Tuba by BestKeeper (Table 3). These genes display the same profile of expression between CR and AL (Fig. 2d). On the other hand, the least stable genes were Gapdh > Hmbs > Pgk1 showed by geNorm, Gapdh > Hmbs > Rpl13a by NormFinder and Gapdh > Pgk1 > Hmbs by BestKeeper (Table 3). While reference genes ranking varied slightly by algorithm, the overall ranking of the best candidate reference genes were calculated through geometric means of the three ranking numbers and the most stable reference genes had the smallest geometric mean^32^,^40^,^41^ (Table 3). The comprehensive ranking showed that β2m and Hprt appear to be the most suitable reference gene pair in iWAT of CR. Besides, the worst reference gene is Gapdh, the most stable one is β2m.

#### In skeletal muscle

The same method was applied in skeletal muscle. All the three algorithms showed that Ppia and Gusb is the best pair of reference genes to normalize RT-qPCR data in skeletal muscle after CR treatment (Table 3). There were some differences in the ranking of reference genes between algorithms. However, the comprehensive ranking based on the geometric mean of the ranks by these algorithms^32^,^40^,^41^ showed that Ppia is the most stable gene and Gapdh is the least stable one.

#### In liver

In liver of CR and AL mice (Table 3), according to the geNorm M value, the best three genes were Hmbs > Tbp > β2m. The results of Normfinder analysis showed that the three most stable reference genes were Hmbs > Ppia > β2m. The results of stable genes by BestKeeper were Hprt > Ywhaz > Hmbs > β2m. Integrating the results of all the three algorithms by geometric mean, the best pair of reference genes to normalize RT-qPCR data in liver after CR treatment appears to be Hmbs and β2m and the most stable gene may be Hmbs. On the other hand, the least stable genes were Gusb > Actb > Hprt analyzed by geNorm, Pgk1 > Hprt > Ywhaz by NormFinder and Pgk1 > Rpl13a > Actb by BestKeeper. The comprehensive ranking showed Pgk1 was the most unstable reference gene.

#### The overall stability of each gene in all the investigated tissues

To assess the overall stability of each gene in all the investigated tissues after CR, we calculated the comprehensive ranking by geometric mean of the ranks of each gene in all the tissues (Table 4). The results showed that after CR, Tbp and Ppia were relative stable, whereas Gapdh and Pgk1 were changed most in all these tissues.

### Validation of reference genes selection on the target mRNA relative expression

**Figure 4:**
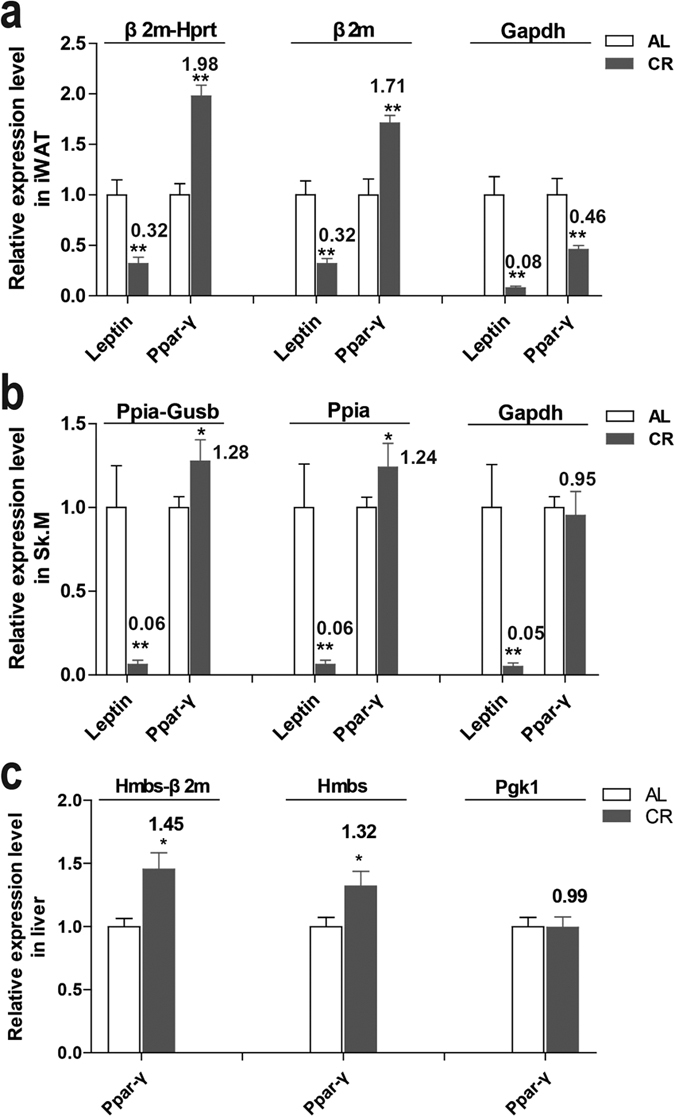
Relative expression level of Leptin and Ppar-γ in inguinal white adipose tissue (**a**), skeletal muscle (**b**) and liver (**c**) of CR and AL mice measured by RT-qPCR and normalized to the indicated reference gene or gene pair. The expression levels of AL mice were set at a relative expression of 1. The fold changes of CR mice were indicated near the bars. PCR reactions for each sample were carried out in triplicate. (*p < 0.05, **p < 0.01 vs AL. n = 5 in each group) AL: ad libitum, CR: caloric restriction.

To validate the reliability of selected reference genes, we decided to check them on the expression of genes which expression changed by CR. In previous studies, CR mice showed a marked increase in Ppar-γ^16^,^42^,^43^ and reduced circulating level of Leptin^15^,^44^,^45^. Leptin is mainly produced by adipocyte^30^,^46^,^47^. Studies have also shown leptin production by other tissues, including skeletal muscle^48^,^49^. Thus, we checked Leptin expression in Leptin- expressing tissues iWAT and Sk.M, and Ppar-γ expression in all the three tissues by normalized with the recommended gene pair, most and least stable gene, respectively (Fig. 4).

By using the recommended reference gene pair by the above analysis - β2m-Hprt in iWAT, Ppia-Gusb in Sk.M and Hmbs-β2 m in liver - we observed the significant decrease of Leptin (p < 0.01) and increase of Ppar-γ (p < 0.01 in iWAT, p < 0.05 in Sk.M and liver) after CR (Fig. 4 left). These data confirmed the reliability of the recommended reference gene pair in each tissue.

The normalization results with the single most stable reference gene - β2m in iWAT, Ppia in Sk.M and Hmbs in liver - were similar to those with gene pair. However, the fold changes of Ppar-γ normalized with these single genes were less than with gene pairs (Fig. 4 middle). On the other hand, when using the least stable reference gene – Gapdh in iWAT and Sk.M and Pgk1 in liver (Fig. 4, right), which are also under the threshold of 1.5 on the geNorm M value - Ppar-γ expression were changed to the opposite direction in iWAT (p < 0.05, Fig. 4a) or had no obvious changes in Sk.M (Fig. 4b) or liver (Fig. 4c) (p > 0.05). These results highlight the importance of selection of suitable reference genes combination and efficient normalization for reliable results in gene expression studies in CR.

## Discussion

In molecular biological research, gene expression analysis is one of the most frequently-used strategies in the field of gene study. RT-qPCR is the most classical technique in the field of gene expression study. This method requires an appropriate reference gene to normalize mRNA levels. It is vital to select appropriate reference genes for normalization of gene expression. Numbers of reference genes have been used, such as Gapdh and Actb, two commonly-used reference genes^9^. However, in many cases, these genes have been demonstrated to be variable in different cells, tissues and experimental conditions as reviewed by Suzuki^9^. For example, Gapdh is not stable in age-induced apoptosis in neurons^27^, in insulin stimulated adipocytes and hepatoma cell lines^22^ and in human omental and subcutaneous adipose tissue from obesity and type 2 diabetes patients^50^.

Therefore, for the first time, we evaluated CR effects in three metabolism related tissues (iWAT, Sk.M and liver) on the expression stability of twelve reference genes (Actb, β2m, Gapdh, Gusb, Hmbs, Hprt, Pgk1, Ppia, Rpl13a, Tbp, Tuba and Ywhaz) by using three different softwares (geNorm, NormFinder and BestKeeper). The most suitable combination of reference genes was recommended for each tissue. We showed that after CR, in all the detected tissues, 1) there are variations of expression for these twelve commonly-used reference genes, 2) at least two reference genes are necessary for good normalization of RT-qPCR data, and 3) each tissue needs its own paired reference genes.

The expression of reference genes can be changed by different experimental conditions or in different tissues^9^,^10^. Tanic and colleagues have detected five housekeeping genes, including Actb, Gapdh, Hprt, 18S rRNA and Cytb, in rat cortex and hippocampus after dietary restriction, a kind of CR. They found that the expression of these genes varied under that condition^19^. It indicates CR can alter some reference genes expression. Their study suggests that it is necessary to evaluate and select appropriate reference genes in CR studies. Furthermore, CR induces extensively responses in all the tissues or organs. There have been quantities of studies on CR, especially in those metabolism related tissues, such as adipose tissues, skeletal muscle and liver^51^,^52^. It has been reported that expression of reference genes in these tissues can also be altered under some conditions. For instance, the work by Zhang *et al*.^53^ found that four frequently-used reference genes have different expression stability in three types of adipose tissue from obese and lean rats. It was also observed by Svingen *et al*. that among the six rat tissues they checked following toxicological exposure, reference genes they detected were most unstable in liver^54^. In pig skeletal muscle, reference genes have variations at multiple developmental stages^55^. However, no study has been carried out to evaluate suitable reference genes in adipose tissues, skeletal muscle and liver after CR. In the present study, for the first time, we detected the effects of CR on reference genes in these tissues. We did find some unstable reference genes after CR in these tissues. Direct comparison of Ct or non-normalized relative quantity revealed significant difference in several genes in each tissue between CR and AL.

Since the instability of reference genes under different experimental conditions, the use of a single, unverified reference gene when normalizing experiments that attempt to demonstrate small differences in mRNA abundance has been shown to lead to unreliable conclusions^6^. By using the recommended pair of reference genes, we observed the same changes of Leptin and Ppar-γ after CR as previously reported^15^,^16^,^42^,^43^,^44^,^45^. It is also reported that CR downregulated^56^ or had no effects on hepatic Ppar-γ expression level^57^,^58^. These inconsistent results of Ppar-γ are possible because of the differences in strains, ages, energy restriction degree, duration, etc. as investigated by Mitchell *et al*.^15^. However, the results normalized with different reference genes can be discrepancy with different extend^29^, have no changes^27^ or even change to opposite direction^59^. In the current study, we observed all these situations when normalized the data with a single reference gene. Although the advantage of using multiple, validated reference genes was demonstrated as early as 2002^7^, until recently, it is still widespread of using single, unverified reference genes^6^, an approach that has been demonstrated to cause biased results. The CR related studies are in the same situation. Our data show that it is important to apply at least two reference genes and to check their stability of expression when they are used to normalize RT-qPCR data.

It is interesting to note that there are some similarities among the three tissues: Gapdh and Pgk1, two metabolism related genes, were changed most in all these tissues, whereas Tbp and Ppia, which are not related with metabolism directly, were ranked low in all the three tissue. It might be due to that they have some similarities in functions or responses, such as they are all insulin target tissues and their insulin sensitivity can all be promoted by CR^39^,^52^. On the other side, it has been well known that gene expression profile has tissue specific. Tissue specificity also happens in reference genes as show in Table 4. Our study showed that each tissue needs its own specific reference genes. For example, β2m is the most stable gene in iWAT and second stable one in liver but relative unstable in Sk.M. Gapdh is most unstable in both iWAT and Sk.M but is ranked in the middle in liver. The facts that these tissues performed different functions and they are all heterogeneous tissues are two possibilities for the differences of reference genes in these tissues.

Considering that an algorithm is one-sided for evaluating the expression stability of reference genes, many statistical approaches are usually integrated to determine the best reference genes in different experimental conditions^27^,^60^. In the present study, we employed three common statistical programs, geNorm, NormFinder, and Bestkeeper. As expected, in each tissue, the stability ranking generated by distinct statistical algorithm is not exactly the same with each other. For example, for Sk.M, Ppia was ranked most stable reference gene by geNorm as well as NormFinder, whereas Gusb was recommended by Bestkeeper. These discrepancies reflect differences between the approaches^7^,^25^,^26^. The geNorm determines gene expression stability based on the principle that the expression ratio of two ideal internal control genes is identical among all test samples^7^. Co-regulated genes with similar expression profiles will obtain preferential stability ranking from geNorm, leading to an erroneous choice for normalization. On the contrary, the algorithms of NormFinder and Bestkeeper are less sensitive to co-regulation^25^,^26^. NormFinder combines both intra- and inter-group variation into a stability value. This model-based approach should provide a more precise and robust estimative of expression variation among subsets composed by different sample types^14^. BestKeeper^15^ software was applied as an expression standard of reference genes according to the ranking of the standard deviation (SD (±Ct)) and coefficient of variance (CV (%Ct)) of Ct values, which is inversely proportional to the stability of expression. However, when there are discrepancies among these methods, the recommended genes are always close to the most stable genes, reflecting their ability to be a good reference gene. It is interesting to note that the discrepancy among different methods is bigger in liver than in iWAT and Sk.M. In liver, Hprt is most stable gene generated by Bestkeeper, but second and third unstable by NormFinder and geNorm. Thus, it is advisable to evaluate reference genes by more than one method and integrate the data to generate final ranking, especially in tissues like liver. Here, we calculated the comprehensive ranking by geometric mean of results by all the software programs^32^,^40^,^41^. And validation by Leptin and Ppar-γ expression assessment confirmed the reliability of this strategy.

In this study we evaluated comprehensively and successfully the reference genes after CR in different tissues. At the same time, we acknowledge the limitations of the present study. First, our choice of reference genes analyzed was limited to twelve candidate genes. There are some other widely used genes, such as 18sRNA and Ubiquitin, which may warrant consideration in the future. However, the selected genes within the twelve candidates are suitable as validated by the expression of Leptin and Ppar-γ. Second, the results of this study may not be applicable to other tissues or treatments. Validation studies will need to be repeated in other tissues, cells, experimental conditions. Nevertheless, according to the similarities among these metabolism related tissues, our results are worthy of reference in metabolism related studies in these tissues. Third, there are some other statistical methods, such as qBasePlus^61^, comparative ΔCt^62^, and RefFinder^63^. We applied only three most commonly-used softwares. However, according to previous studies^64^,^65^, it’s possible that those methods could get similar results as in this study.

## Conclusion

This is the first study specifically designed to evaluate effects of CR in iWAT, skeletal muscle and liver on a set of candidate reference genes for gene expression normalization using qRT-PCR in the C57BL6/J mice using the programs geNorm, NormFinder, and BestKeeper. We indicate that among 12 candidate reference genes investigated, each tissue needs its own combination of reference genes, which is β2m-Hprt for iWAT, Ppia-Gusb for Sk.M and Hmbs-β2m for liver. We also demonstrate the advantage of using more than one reference gene in combination for certain experimental conditions. Our study provides a basis for the selection of reference genes and useful guidelines for future gene expression studies of CR and metabolism.

## Additional Information

**How to cite this article**: Gong, H. *et al*. Evaluation of candidate reference genes for RT-qPCR studies in three metabolism related tissues of mice after caloric restriction. *Sci. Rep.* **6**, 38513; doi: 10.1038/srep38513 (2016).

**Publisher's note:** Springer Nature remains neutral with regard to jurisdictional claims in published maps and institutional affiliations.

## Supplementary Material

Supplementary Information

## Figures and Tables

**Table 1:** Gene symbols, gene names, accession numbers and functions of the twelve reference genes investigated by RT-qPCR.

Gene Symbol	Gene Name	Accession Number	Function
Actb	beta-actin	NM_007393.5	Cytoskeletal structural protein
β2m	beta-2 microglobulin	NM_009735.3	Beta-chain of MHC class I molecules
Gapdh	glyceraldehyde-3-phosphate dehydrogenase	NM_008084.3	Involved in glycolysis and gluconeogenesis
Gusb	beta-glucuronidase	NM_010368.1	Lysosomal exoglycosidase
Hmbs	hydroxymethylbilane synthase	NM_013551.2	Heme synthesis and porphyrin metabolism
Hprt	hypoxanthine guanine phosphoribosyl transferase	NM_013556.2	Purine synthesis through the purine salvage pathway
Pgk1	phosphoglycerate kinase 1	NM_008828.3	A glycolytic enzyme
Ppia	peptidylprolyl isomerase A	NM_008907.1	Protein coding, a cyclosporin binding-protein
Rpl13a	ribosomal protein L13A	NM_009438.5	Sturcturalconstituent of ribosome
Tbp	TATA box binding protein	NM_013684.3	Transcription initiation from RNA polymerase II, general transcription factor
Tuba	alpha-tubulin	NM_011653.2	Microtubules of the eukaryotic cytoskeleton
Ywhaz	tyrosine 3-monooxygenase/tryptophan 5-monooxygenase activation protein, zeta-polypeptide	NM_011740.3	Protein domain in specific binding

**Table 2:** List of reference genes investigated by RT-qPCR.

Gene Symbol	Primer Sequence	Amplicon Size	Span Intron	Efficiency%	R^2^
Actb	F:CCTTCTTGGGTATGGAATCCTGT R:CACTGTGTTGGCATAGAGGTCTTTAC	101	Y (E4-E5)	98	0.9997
β2m	F:CATGGCTCGCTCGGTGAC R:CAGTTCAGTATGTTCGGCTTCC	135	Y (E1-E2)	101	0.9994
Gapdh	F:TGCACCACCAACTGCTTAG R:GGATGCAGGGATGATGTTC	177	Y (E4-E5)	101	0.9999
Gusb	F:CCGACCTCTCGAACAACCG R:GCTTCCCGTTCATACCACACC	169	Y (E1-E2)	104	0.9992
Hmbs	F:ATGAGGGTGATTCGAGTGGG R:TTGTCTCCCGTGGTGGACATA	134	Y (E2-E4)	99	0.9999
Hprt	F:TGACACTGGCAAAACAATGCA R:GGTCCTTTTCACCAGCAAGCT	95	Y (E6-E7)	107	1
Pgk1	F:ATGTCGCTTTCCAACAAGCTG R:GCTCCATTGTCCAAGCAGAAT	164	Y (E1-E3)	99	0.9948
Ppia	F:GGCAAATGCTGGACCAAAC R:CATTCCTGGACCCAAAACG	149	Y (E4-E5)	105	0.9996
Rpl13a	F:AGGGGCAGGTTCTGGTATTG R:TGTTGATGCCTTCACAGCGT	120	Y (E1-E3)	101	0.9989
Tbp	F:CCTTGTACCCTTCACCAATGAC R:ACAGCCAAGATTCACGGTAGA	119	Y (E3-E4)	96	0.9913
Tuba	F:TGTCCTGGACAGGATTCGC R:CTCCATCAGCAGGGAGGTG	115	Y (E3-E4)	104	1
Ywhaz	F:GAAAAGTTCTTGATCCCCAATGC R:TGTGACTGGTCCACAATTCCTT	134	Y (E3-E4)	101	0.9999

Primers sequences, length of the RT-qPCR transcripts, efficiency of each pair of primers and the correlation coefficients (R^2^) of the standard curve are indicated.

**Table 3:** Candidate reference gene expression stability ranked by geNorm, NormFinder, and BestKeeper in iWAT, Sk.M and liver of CR mice.

Tissue	Rank	geNorm		NormFinder		BestKeeper			Comprehensive ranking by geoMean
		Gene	M	Gene	Stability value	Gene	CV (% Ct)	SD (±Ct)	
iWAT	1	Gapdh	1.024	Gapdh	0.616	Gapdh	4.158	0.869	Gapdh
	2	Hmbs	0.694	Hmbs	0.400	Pgk1	2.498	0.492	Hmbs
	3	Pgk1	0.653	Rpl13a	0.340	Hmbs	2.442	0.555	Pgk1
	4	Actb	0.579	Pgk1	0.323	Actb	2.330	0.483	Actb
	5	Rpl13a	0.557	Actb	0.321	Gusb	2.197	0.493	Rpl13a
	6	Gusb	0.547	Ywhaz	0.286	Rpl13a	2.173	0.389	Gusb
	7	Ywhaz	0.505	Gusb	0.278	Ywhaz	1.620	0.313	Ywhaz
	8	Tuba	0.490	Tuba	0.163	Tbp	1.542	0.380	Tuba
	9	Hprt	0.460	Tbp	0.134	Ppia	1.527	0.263	Tbp
	10	Ppia	0.447	Hprt	0.134	Tuba	1.505	0.332	Ppia
	11	Tbp	0.446	Ppia	0.129	β2m	1.484	0.323	Hprt
	12	β2m	0.419	β2m	0.104	Hprt	0.847	0.175	β2m
Sk.M	1	Gapdh	0.437	Gapdh	0.232	Gapdh	1.657	0.290	Gapdh
	2	Hmbs	0.380	Hprt	0.189	Ywhaz	1.187	0.288	Hprt
	3	Hprt	0.374	Rpl13a	0.172	Tuba	1.070	0.246	Tuba
	4	Pgk1	0.347	Tuba	0.171	Actb	1.035	0.226	Ywhaz
	5	β2m	0.345	Ywhaz	0.169	β2m	1.018	0.250	β2m
	6	Rpl13a	0.343	β2m	0.158	Hprt	0.968	0.250	Pgk1
	7	Tuba	0.334	Pgk1	0.155	Pgk1	0.953	0.206	Rpl13a
	8	Tbp	0.332	Tbp	0.153	Hmbs	0.855	0.227	Hmbs
	9	Ywhaz	0.327	Actb	0.152	Rpl13a	0.750	0.168	Actb
	10	Actb	0.324	Hmbs	0.151	Tbp	0.690	0.206	Tbp
	11	Gusb	0.268	Gusb	0.057	Ppia	0.511	0.121	Gusb
	12	Ppia	0.251	Ppia	0.040	Gusb	0.357	0.099	Ppia
Liver	1	Gusb	0.414	Pgk1	0.204	Pgk1	1.776	0.341	Pgk1
	2	Actb	0.392	Hprt	0.204	Rpl13a	1.624	0.298	Actb
	3	Hprt	0.384	Ywhaz	0.200	Actb	1.483	0.288	Gusb
	4	Tuba	0.372	Tuba	0.158	Gapdh	1.405	0.258	Hprt
	5	Pgk1	0.370	Gapdh	0.153	Ppia	1.090	0.193	Tuba
	6	Ywhaz	0.368	Actb	0.151	Tuba	1.069	0.236	Rpl13a
	7	Rpl13a	0.343	Gusb	0.150	Gusb	1.016	0.268	Gapdh
	8	Gapdh	0.325	Rpl13a	0.139	Tbp	0.983	0.245	Ywhaz
	9	Ppia	0.307	Tbp	0.132	β2m	0.767	0.147	Ppia
	10	β2m	0.306	β2m	0.115	Hmbs	0.611	0.133	Tbp
	11	Tbp	0.304	Ppia	0.083	Ywhaz	0.602	0.137	β2m
	12	Hmbs	0.276	Hmbs	0.059	Hprt	0.579	0.141	Hmbs

The comprehensive ranking was based on the geometric mean of geNorm, NormFinder, and BestKeeper results. Candidates are listed from top to bottom in order of increasing expression stability. (SD (±Ct): standard deviation of the Ct; CV (% Ct): coefficient of variance expressed as a percentage of the Ct level; iWAT: inguinal white adipose tissue; Sk.M: skeletal muscle; geoMean: geometrical mean).

**Table 4:** The comprehensive ranking of the candidate reference gene expression stability after CR.

Gene	Comprehensive ranking by GeoMean	Rank in iWAT	Rank in Sk.M	Rank in liver
Gapdh	1	1	1	7
Pgk1	2	3	6	1
Actb	3	4	9	2
Hprt	4	11	2	4
Tuba	5	8	3	5
Hmbs	6	2	8	12
Gusb	7	6	11	3
Rpl13a	8	5	7	6
Ywhaz	9	7	4	8
β2m	10	12	5	11
Tbp	11	9	10	10
Ppia	12	10	12	9

The comprehensive ranking was based on the geometric mean of the gene rank in each tissue. Candidates are listed from top to bottom in order of increasing expression stability. (iWAT: inguinal white adipose tissue; Sk.M: skeletal muscle; geoMean: geometric mean).
