# Supplementary material for: Evaluation of candidate reference genes for RT-qPCR studies in three metabolism related tissues of mice after caloric restriction
Source: Sci Rep. 2016 Dec 6;6:38513. doi: 10.1038/srep38513 (PMC5138604; doi:10.1038/srep38513)
Supplement: Supplementary Information [file srep38513-s1.pdf]

Evaluation of candidate reference genes for RT-qPCR studies in three metabolism related tissues of mice after caloric restriction

Huan Gong, Liang Sun*, Beidong Chen, Yiwen Han, Jing Pang, Wei Wu, Ruomei Qi, and Tie-mei Zhang*

The MOH key laboratory of Geriatrics, Beijing Hospital, National Center of Gerontology, Beijing, P.R.China.

*Corresponding author: Tie-mei Zhang and Liang Sun

Beijing Institute of Geriatrics, Beijing Hospital, No.1 Dahua Road, Dong Dan, Beijing 100730, P. R. China.

Email: tmzhang126@126.com, sunbmu@foxmail.com

Tel: 86-10-58115044, 86-10-58118043

Fax: 86-10-65237929

Supplementary Fig. S1

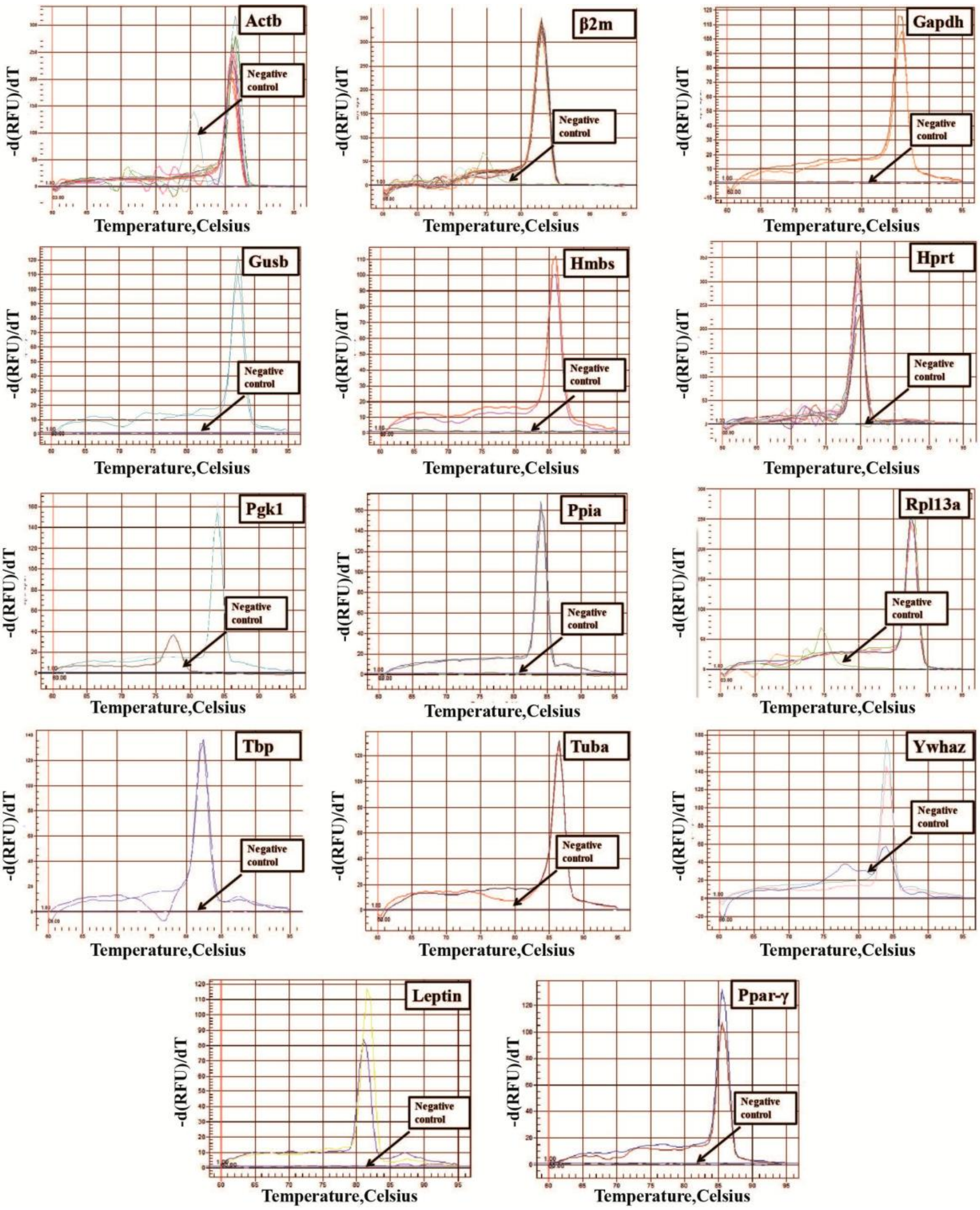

Fig. S1 **Melting curves of the qRT-PCR products.** The single sharp peak represents a specific qRT-PCR product. The first twelve genes, including Actb, β2m, Gapdh, Gusb, Hmbs, Hprt, Pgk1, Ppia, Rpl13a, Tbp, Tuba, and Ywhaz are candidate reference genes. The last two genes, including Leptin and Ppar-γ are used for validation of the selected reference genes. Negative control is the melting curve of the qPCR product of reverse transcription negative control.

Supplementary Fig. S2

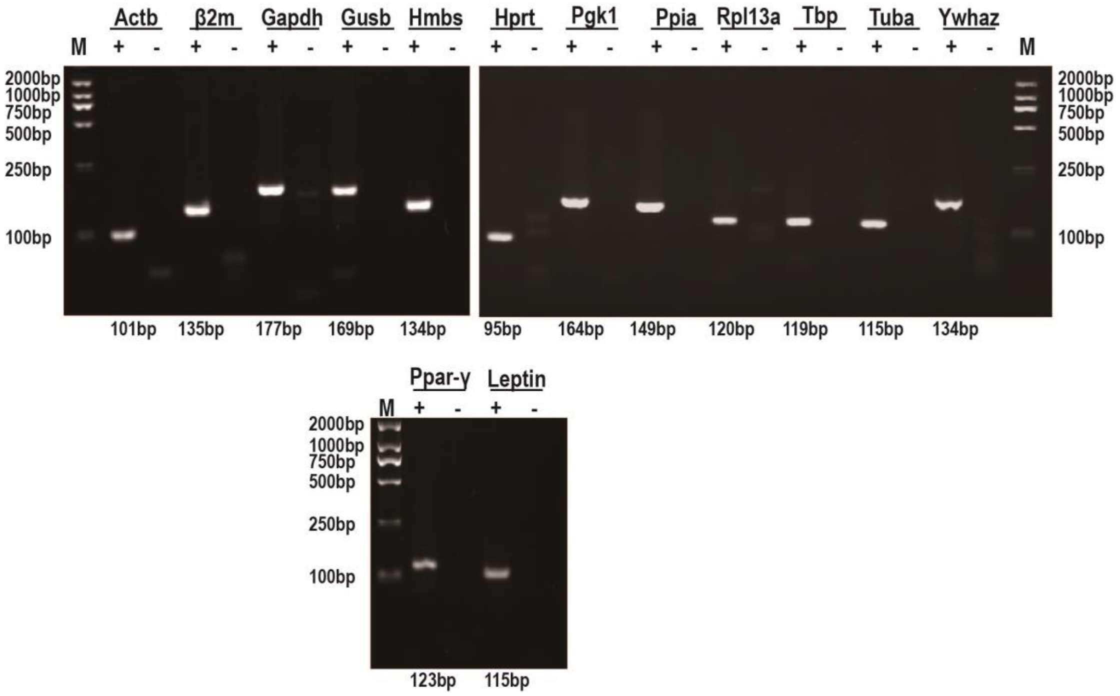

Fig. S2 **Agarose gel electrophoresis of the qRT-PCR products.** The single sharp band represents a specific qRT-PCR product. Length of each qRT-PCR product is labeled under each band. Actb, β2m, Gapdh, Gusb, Hmbs, Hprt, Pgk1, Ppia, Rpl13a, Tbp, Tuba, and Ywhaz are candidate reference genes, Ppar-γ and Leptin are used for validation. M: DNA ladder, +: positive sample, -: reverse transcription negative control.

Supplementary Fig. S3

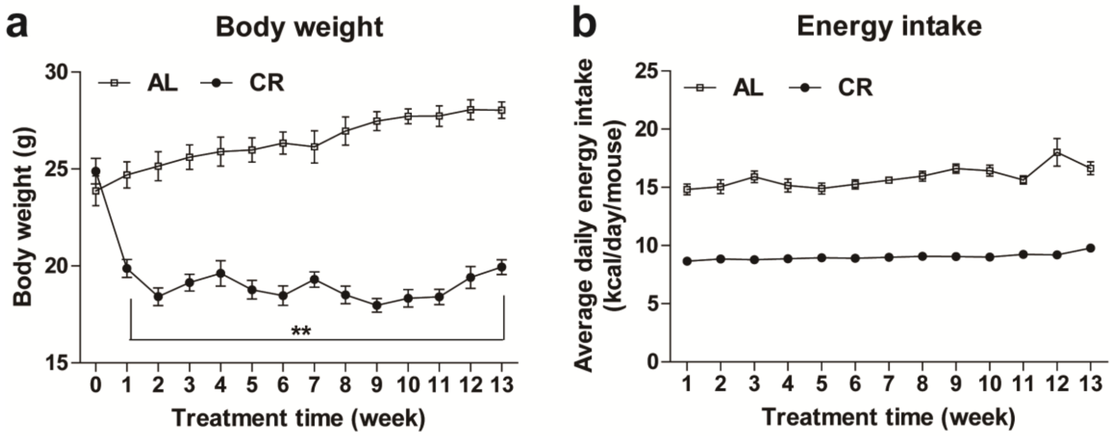

Fig. S3 **The body weight and the average daily energy intake of mice in AL or CR group.** (a) The average body weight of mice from the two groups in each week. (b) The average daily energy

intake of mice from the two groups in each week. The food intake was measured once a week. (* *: $p < 0.01$ versus AL group. $n = 5$ in each group) AL: ad libitum, CR: caloric restriction.
